# Metabolic Syndrome and Cognitive Functions in Schizophrenia—Implementation of Dietary Intervention

**DOI:** 10.3389/fpsyt.2020.00359

**Published:** 2020-04-30

**Authors:** Katarzyna Adamowicz, Aleksandra Mazur, Monika Mak, Jerzy Samochowiec, Jolanta Kucharska-Mazur

**Affiliations:** ^1^Department and Clinic of Psychiatry, Pomeranian Medical University, Szczecin, Poland; ^2^Department of Social Sciences, Institute of Psychology, University of Gdańsk, Gdańsk, Poland

**Keywords:** schizophrenia, metabolic syndrome, cognitive functions, Mediterranean diet, dietary intervention

## Abstract

**Introduction:**

The co-existence of schizophrenia and metabolic syndrome is a widely described phenomenon that contributes to the worse functioning of patients in everyday life. A relatively new area of research is the relationship between metabolic syndrome (MS) and cognitive function in patients with schizophrenia. The aim of the study was to verify the relationship between the presence of metabolic syndrome and cognitive function of patients with schizophrenia and to assess the possibility of changing cognitive function by introducing appropriate dietary intervention.

**Materials and Methods:**

The study involved 87 individuals diagnosed with schizophrenia according to ICD-10 criteria, aged 19 to 67 years (M = 41.67; SD = 11.87). Patients were in the remission phase of schizophrenia, all using antipsychotics for pharmacological treatment. From a group of 83 patients with schizophrenia and diagnosed metabolic syndrome (according to IDF criteria) 30 patients were randomly assigned to an experimental group—with dietary intervention, 29 patients—to group without dietary intervention, 24 patients with schizophrenia without metabolic syndrome was a comparison group. All groups were evaluated for cognitive function using Stroop Test, Trail Making Test (TMT), Verbal Fluency Test, Digit Span Backwards Test. In the experimental group a dietary intervention was applied, which was to provide the examined person with a 7-day dietary plan with reduced calorie content, in compliance with the Mediterranean diet.

**Results:**

After the dietary intervention there was a significant improvement in the number of errors made in the third Stroop Test (p <0.001), the time taken to complete the Point Linking Test was shortened (Test B; p = 0.005), there was an improvement in Verbal Fluency Test in “animals” category (p = 0.006) “sharp objects” category (p = 0.009), the number of repeated digits has increased in Digit Span Test in “forward” category (p = 0.001) and overall completion of the test (p = 0.021). In the group of patients with MS without dietary intervention, the results of cognitive tests remained mostly unchanged.

**Conclusions:**

Change of eating habits may be a significant element of a holistic approach to the problems of treatment of schizophrenia.

## Introduction

The coexistence of schizophrenia and metabolic syndrome is widely described in medical literature. Mental illness alone significantly impairs the functioning of the patient, and the occurrence of somatic disease further reduces the quality of life and functioning. It is also worth noting that about 50% of patients with mental disorders have at least one undiagnosed somatic disease (1). Therefore, it is worthwhile to address topics concerning the co-occurrence of mental disorders and somatic diseases.

In schizophrenia, cognitive dysfunctions (especially executive dysfunctions) are common. They usually appear already in childhood, most often they concern memory, visual-motor coordination and attention (2). Cognitive symptoms are an equally important element of the disorder as positive, negative and affective symptoms. Each of psychotic symptom clusters is associated with and may contribute to worsening of other symptoms clusters, worsening the patient’s condition, in a similar manner to coexisting somatic diseases. As a result, the activity in specific areas of individual functioning is reduced (2–4).

A relatively new direction of research is the coexistence of metabolic syndrome and cognitive disorders in patients with schizophrenia. Meta-analysis of 18 studies confirms that the presence of MS (metabolic syndrome) or its components is associated with significantly worse cognitive function (5). Obesity is one of the components of MS, which causes a higher risk of developing neuropsychiatric disorders. On the other hand, patients with mental illnesses are more prone to unhealthy eating habits, and pharmacological treatment increases appetite, which may contribute to the development of obesity. The secretion of neuropeptide Y (NPY) is responsible for the appearance of the feeling of hunger. Studies show that when pharmacotherapy is included, an increase in concentration of leptin is observed, which entails incorrect NPY secretion and causes and increased need to consume food. Excessive supply of energy from food causes overweight and obesity to appear later on. A change in the production of hormones that regulate appetite can already be observed in the initial phase of psychotic disorders. It has also been shown that compared to people without schizophrenia, those diagnosed with schizophrenia have three times more visceral adipose tissue (6, 7). A meta-analysis of 48 studies showed that olanzapine is a drug that causes the greatest weight gain and increase in cholesterol. Large variations in weight gain are also observed when using clozapin. However, aripiprazole, risperidone or ziprasidone seem to be safer. When choosing the right neuroleptic, it is necessary to take into account not only its antipsychotic effect, but also its influence on the occurrence of metabolic side effects (8).

Both obesity and schizophrenia are associated with systemic inflammation. Obesity is often accompanied by neuroendocrine changes associated with the functioning of the hypothalamic–pituitary–adrenal axis. Moreover, obesity-related stress increases the risk of episodes of mood disorders, exacerbation of psychosis and deterioration of cognitive functions (9, 10). It has been noted that MS is a risk factor for the development of cognitive disorders, as obesity, hypertension and hyperglycemia are associated with inflammation of the nervous system, leading to cognitive impairment (11–13). Boyer et al. observed that patients with schizophrenia and concomitant MS have worse results in tests examining executive functions, attention and memory than patients with schizophrenia but without MS (14). It was also noted that a high BMI may contribute to a deterioration in memory, language functions, performance and even general intelligence (15). People with mental illnesses, with excessive fatty tissue content, perform worse in tests checking deferred memory, direct memory, and have reduced efficiency in abstract thinking (16). There were also connections between the occurrence of metabolic syndrome in adulthood and reduced cognitive function during adolescence, in people who were diagnosed with schizophrenia in adulthood. The study noted that poor school performance between the ages of 12 and 16 was linked to the emergence of MS in the future. This suggests that in addition to antipsychotics, the cognitive factor may be responsible for the risk of metabolic disorders later in life. Therefore, health education programs should be introduced during adolescence, especially for people with impaired cognitive function (17, 18).

Of course, the metabolic syndrome itself, without accompanying schizophrenia, may negatively affect cognitive functions. This was observed in middle-aged people with a diagnosis of MS, without mental illness, who, in comparison to their peers without MS, have greater problems in psychomotor efficiency and speed, creating concepts, decision making and intellectual functioning (19). Individuals with pathological obesity, in comparison to the group with BMI in normal range, obtain worse results in terms of operating memory and executive functions. They also show higher rigidity of thinking, which indicates the occurrence of cognitive dysfunctions related to prefrontal cortex function (20). The use of magnetic resonance imaging has shown that obese people have a smaller volume of white and grey matter in temporal and frontal structures compared to people with a normal body weight. The high adipocyte content in an obese person’s body may amplify the cellular inflammatory response, which in turn damages brain tissue, especially oligodendrocytes, and, as a result, impairs cognitive functions (21–23).

On the other hand, actions aimed at improving the components of MS may improve cognitive efficiency (24). Discovering further links between cognitive function and dietary behaviors, which have a significant influence on the development of MS, may contribute to understanding the mechanisms of obesity formation. The relationship between obesity and cognitive disorders may be bilateral. Loss of control over food intake is associated with changes in cognitive efficiency (25). Lips et al. found that fasting obese people show stronger functional communication between the brain areas responsible for cognitive control, motivation and reward system than people with normal body weight (26). Neural network connections in lean and obese individuals were also examined by means of functional magnetic resonance imaging. Functional integrity of resting network connections is different in both groups, especially in default mode network and frontal lobe network. This confirms that obesity affects brain function in areas responsible for nutrition regulation or reward system (27). These studies show that in planning the therapy for schizophrenia, both psychiatric and medical treatment, including dietary management, should be taken into account.

The aim of this study was to verify the relationship between the presence of metabolic syndrome and cognitive function of patients with schizophrenia and to assess the possibility of changing cognitive function by introducing appropriate dietary intervention. The following research hypotheses were posed:

1. Cognitive function of patients with schizophrenia and coexisting metabolic syndrome is reduced in comparison to patients with schizophrenia without metabolic syndrome.
2. The implementation of appropriate dietary intervention contributes to the improvement of cognitive function in patients with schizophrenia and coexisting metabolic syndrome.

The presented study is a part of a larger project concerning eating habits and metabolic syndrome in patients with schizophrenia. The study was approved by the Bioethics Committee of the Pomeranian Medical University in Szczecin (Resolution No. KB-0012/108/14).

## Materials and Methods

### Participants

The study involved 87 patients with schizophrenia (F 20 according to ICD 10) in remission lasting at least 3 months at the time of giving consent to participate in the study, treated outpatient in the Department of Psychiatry of the Pomeranian Medical University in Szczecin. Four patients were excluded due to significant lack of data in the second measurement (lack of at least one test). Therefore, the analysis was carried out on 83 patients with F 20 diagnosis, aged from 19 to 67 years, of whom 59 (71.08%) met the diagnostic criteria of the metabolic syndrome.

### Clinical Assessment

After obtaining written consent to participate in the study, each patient was examined by a specialist psychiatrist to verify the diagnosis of schizophrenia and to establish that the patient is in remission of the disease (using PANSS). The study also included an assessment of somatic status to exclude serious diseases that may affect cognitive functions (severe CNS damage, stroke, liver, kidney or heart failure, cancer, significant anemia, endocrine disorder, addiction to psychoactive substances excluding nicotinism). Patients who were diagnosed with these diseases were excluded from the study. The drugs used to treat the patients were olanzapine, clozapine, quetiapine and aripiprazole. Each patient has used at least one of these drugs. There were no statistically significant differences in pharmacological treatment, except for aripiprazole [X2(2) = 15.574; p < 0.001], 50% (n = 12) of patients without MS took this drug, while in the group with MS 23.3% (n = 7) and without dietary intervention 3.4% (n = 1).

### Implements

Metabolic syndrome is associated with the coexistence of various risk factors that can cause cardiovascular disease and type 2 diabetes. There are no commonly accepted criteria for metabolic syndrome, but it is generally acknowledged to include abdominal obesity, glucose intolerance, hypertension and dyslipidemia. In order to determine the presence of metabolic syndrome according to IDF (International Diabetes Federation) criteria from 2005, anthropometric measurements, blood pressure measurement and laboratory tests were used (28). All patients had anthropometric measurements taken, which included measurements of height, body weight, hip circumference, waist circumference. Height was measured using a height gauge with an accuracy of ±0.1 cm with a fully upright posture, without shoes. Body weight was measured using an electronic scale with an accuracy of ±0.1 kg. In all patients the following blood levels were determined: total cholesterol, LDL, HDL, triglycerides and glucose. The obtained results were referenced to baseline values according to the recommendations of PTKK (Polish Society of Cardiology) and PTDL (Polish Society of Laboratory Diagnostics). A prerequisite was the occurrence of abdominal obesity (i.e. waist circumference of >94 cm in men and >80 cm in women), and any two of the following: raised triglycerides (> 150 mg/dl); reduced HDL cholesterol (< 40 mg/dl in men, < 50 mg/dl in women); raised blood pressure (> 130/85 mmHg); raised fasting plasma glucose (> 100 mg/dl) (29).

Four tests were used for assessment of cognitive function:

1. Stroop—used to check the reading speed, verbal operational memory and execution functions related to inhibiting the habitual response and the ability to switch to a new response criterion (30, 31),
2. Trail Making Test (TMT)—enables for the evaluation of visual-spatial operational memory, visual-motor coordination and psychomotor speed. Polish adaptation of the whole Halstead–Reiten battery under the supervision of Danuta Kądzielawa (32, 33),
3. Verbal Fluency—checks categorical verbal fluency (semantic) and phonemic fluency (literal) (34),
4. Digit Span Backwards—tests memory storage capabilities and numerical skills associated with the functioning of operational verbal memory (35).

After obtaining the above information, patients from the group with schizophrenia and MS were randomly assigned to the group with dietary intervention (MSwithDI) or without dietary intervention (MSw/oDI). The exact characteristics of the sample in terms of sociodemographic features and PANSS scale results, including the division of patients with MS into experimental and control groups, are presented in **Table 1**. The groups differed slightly in the distribution of the place of residence—among all participants of the study most individuals were from very large cities, but in the MSwithDI subgroup the advantage was slightly greater. (p < 0.05).

**Table 1:** Sociodemographic characteristics of groups studied, N(%) or M(SD).

Variables	Entire group, N = 83	MSwithDI, n = 30	MSw/oDI, n = 29	SH, n = 24	X^2^/F	df	P
Age	41.06 (12.82)	44.33 (12.38)	41.66 (13.32)	36.25 (11.76)	2,846	2	0.066
Sex							
Female	53 (63.9%)	20 (66.7%)	16 (55.2%)	17 (70.8%)	1,556	2	0.459
Male	30 (36.1%)	10 (33.3%)	13 (44.8%)	7 (29.2%)			
Education					4,566	6	0.601
Primary	6 (7.2%)	1 (3.3%)	2 (6.9%)	3 (12.5%)			
Vocational	17 (20.5%)	6 (20.0%)	8 (27.6%)	3 (12.5%)			
Secondary	37 (44.6%)	13 (43.3%)	11 (37.9%)	13 (54.2%)			
Higher	23 (27.7%)	10 (33.3%)	8 (27.6%)	5 (20.8%)			
Place of residence							
Village	13 (15.7%)	4 (13.3%)	5 (17.2%)	4 (16.7%)	15,707	8	0.047
Town	1 (1.2%)	0 (0.0%)	0 (0.0%)	1 (4.2%)			
City	7 (8.4%)	2 (6.7%)	1 (3.4%)	4 (16.7%)			
Large city	5 (6.0%)	0 (0.0%)	5 (17.2%)	0 (0.0%)			
Very large city	57 (68.7%)	24 (80.0%)	18 (62.1%)	15 (62.5%)			
Job market status							
Student	21 (25.3%)	7 (23.3%)	10 (34.5%)	4 (16.7%)	8,143	6	0.228
Employed	4 (4.8%)	0 (0.0%)	1 (3.4%)	3 (12,5%)			
Unemployed	12 (14.5%)	3 (10.0%)	5 (17.2%)	4 (16.7%)			
Retired/Pensioner	4 (55.4%)	20 (66.7%)	13 (44.8%)	13 (54.2%)			
Marital status							
Single	39 (47.0%)	11 (36.7%)	14 (48.3%)	14 (58.3%)	4,137	6	0.658
In a relationship	32 (38.6%)	13 (43.4%)	11 (37.9%)	8 (33.3%)			
Divorced	7 (8.4%)	3 (10.0%)	2 (6.9%)	2 (8.3%)			
Widow/Widower	5 (6.0%)	3 (10.0%)	2 (6.9%)	0 (0.0%)			
PANSS							
Positive	8.24 (1.83)	8.23 (1.41)	8.69 (2.49)	7.71 (1.12)	1,942	2	0.150
Negative	11.69 (4.04)	11.53 (3.95)	12.62 (4.39)	10.75 (3.59)	1,461	2	0.238
General	20.88 (4.75)	20.73 (4.09)	21.97 (5.28)	19.75 (4.74)	1,470	2	0.236

Source: own research.

When planning dietary intervention, the Standards of Dietary Therapy of Simple Obesity in adults were used according to the recommendations of the Polish Dietetic Association issued in 2015 (36). The patient was given a 7-day menu. Two versions of dietary plans were prepared. In women, the calorific value of the proposed diet ranged from 1,500 to 1,700 kcal, while in men 2,000 to 2,200 kcal was estimated, with permitted minor deviations of 100 to 200 kcal, due to the fact that each time individual needs and preferences of the patient were considered concerning products that would be acceptable to them in the proposed dietary plan and minor modifications to the diet were made in accordance with them. This was done in order to make it easier for the patient to follow the proposed changes in their eating habits. In the proposed dietary plan, appropriate proportions of macro-elements were also established in relation to total dietary energy—protein 10–25%, carbohydrates 45–65%, fat 20–35% (37). The main assumptions of the prepared diet were as follows:

- reduction of the calorific value of the meal plan in relation to the total demand,
- complete elimination of sweets,
- the introduction of the desired culinary techniques,
- diversification of the existing diet,
- consumption of fruit and vegetables,
- introduction of products rich in desirable fatty acids, e.g. fish, avocados, etc,
- regularity of food consumption.

The prepared menus were based on the principles of the Mediterranean diet (38). The nutrition plan for patients was prepared with the use of the ALIANT computer program—a dietary calculator. Each day of the proposed diet included five meals per day, which were eaten at specific times after being agreed with the participant. The meal plan was used by patients for 3–4 months after receiving it, followed by a control visit, during which cognitive function was checked again by means of the above mentioned cognitive tests. After the same period, the cognitive performance of patients without dietary intervention was also checked, both without and with MS. Dietary compliance was monitored on the basis of statements made by the patients themselves and objective interviews with their relatives, in the meantime, changes in anthropometric measurements were checked. Patients from both groups were also evaluated psychiatrically to assess whether the remission of schizophrenia persisted and whether their somatic status deteriorated. There was no change in treatment in patients throughout the study.

### Statistics

Quantitative data analysis was performed with the use of a statistical software IBM SPSS Statistics v.25. Shapiro–Wilk test was used to assess the compliance of quantitative characteristics distribution with the normal distribution and Pearson’s Chi-square test to assess differences in distributions of qualitative variables. The first of the research hypotheses was checked with two methods—to assess intergroup differences in quantitative test results, the analysis of variance using the ANOVA one-way method was used, with an additional correction by Brown–Forsyth, due to partial deviations from the normal distribution, for comparison. After the assessment of the homogeneity of variances using Levene’s test, Tukey’s HSD was used as a post-hoc test, provided that the variance was homogeneous and Dunnet’s T3 when there was no homogeneity. To determine the differences in the number of errors made in the tests, the non-parametric equivalent of ANOVA, i.e. the Kruskal–Wallis Test, was used. The second hypothesis was verified with the Student’s t test for two dependent groups, when the differences between the first and second measurement in quantitative results of cognitive tests were checked, as well as the non-parametric Wilcoxon test to assess the differences in the number of errors made. The significance index was calculated as p < 0.05 and p < 0.1 as an indicator of not fully significant statistical tendency.

## Results

All three groups (patients with schizophrenia without metabolic syndrome (SH), patients with schizophrenia and metabolic syndrome without dietary intervention (MSw/oDI), patients with schizophrenia and metabolic syndrome undergoing dietary intervention (MSwithDI) did not differ in the initial results of the cognitive function study (p > 0.05). **Table 2** contains information about the minimum, maximum and mean results obtained by patients and the significance of deviations of data distribution from normal distribution (marked with “*” symbols). Since the differences in the number of three compared subgroups were not statistically significant [X2(2) = 0.707; p = 0.688], a parametric method of variance analysis was used to assess the differences in the intensity of quantitative results.

**Table 2:** Results of cognitive function and results allowing for diagnosis of metabolic syndrome measurement before dietary intervention; min–max; M (SD).

Tools	Task	MSwithDI, n = 30	MSw/oDI, n = 29	SH, n = 24	One-way ANOVA		
					F	df	p
**Stroop Test**	**Trial 1—time [s]**	15.45–35.50; 22.46 (5.55)^**^	15.81–71.52; 24.80 (10.50)_***_	17.81–35.79; 22.84 (4.73)^***^	0.844	2	0.436
	**Trial 2—time [s]**	19.67–39.51; 28.64 (5.01)	17.20–86.83; 32.72 (13.70)^***^	21.62–59.31; 32.09 (9.22)^***^	1.416	2	0.251
	**Trial 3—time [s]**	34.61–67.24; 52. 36 (8.44)	28.77–137.42; 57.53 (19.84)^***^	36.69–103.71; 60.16 (18.63)^**^	1.578	2	0.215
**Trail Making Test**	**Test A [s]**	20.52–112.88; 47.47 (21.64)^***^	23.57–121.26; 46.52 (21.90)^***^	23.04–97.84; 45.89 (19.20)^**^	0.039	2	0.962
	**Test B [s]**	39.32–272.25; 108.67 (50.90)^*^	39.98–207.43; 94.60 (44.13)^**^	52.93–154.26; 97.11 (27.84)	0.947	2	0.393
**Verbal Fluency Test**	**Animals**	5.00–23.00; 15.00 (4.23)	3.00–27.00; 14.66 (5.49)	5.00–21.00; 14.67 (4.03)	0.052	2	0.949
	**Objects starting with letter “K”**	3.00–13.00; 8.07 (2.95)	0.00–17.00; 7.52 (4.21)	3.00–15.00; 7.42 (3.22)	0.283	2	0.755
	**Sharp objects**	1.00–4.00; 6.33 (3.12)^*^	1.00–13.00; 6.35 (2.47)	3.00–13.00; 6.92 (2.67)	0.373	2	0.690
**Digit Span Test**	**Forward**	3.00–8.00; 5.70 (1.21)^*^	3.00–13.00; 6.21 (2.14)^**^	4.00–9.00; 5.54 (1.53)^***^	1.184	2	0.312
	**Backward**	2.00–8.00; 4.40 (1.67)^**^	2.00–9.00; 4.45 (1.66)^**^	2.00–8.00; 4.42 (1.56)	0.007	2	0.993
	**All**	6.00–15.00; 10.10 (2.41)	6.00–22.00; 10.57 (3.24)^***^	4.00–17.00; 9.79 (2.93)	0.517	2	0.599
**MS components**	**Waist circumference**	94.00–148.00 113.77 (14.97)	93.00–160.00 110.63 (13.90)^**^	72.00–121.00 94.15 (12.40)	16.379	2	<0,001
	**Body mass**	73.00–147.70 101,24 (19,65)	67.00–129.00 93.00 (16.37)	44.00–112.00 76.98 (15.59)	14.377	2	<0,001
	**BMI**	27.14–48.88 35.09 (5.55)^*^	24.81–44.12 32.09 (4.86)^*^	19.56–39.68 27.74 (4.71)	15.160	2	<0,001
	**Glucose**	80.00–399.00 117.22 (62.33)^***^	61.50–231.00 109.09 (38.29)^***^	74.10–231.20 97.59 (29.88)^***^	1.336	2	0,270
	**HDL**	23.20–81.50 44.76 (14.63)^*^	21.70–69.44 39.66 (12.74)	35.00–74.80 56.77 (10.29)	13.612	2	<0,001
	**TG**	71.70–455.00 209.42 (86.75)^**^	68.30–653,40 246,09 (133.87)^*^	44.00–157.00 90.83 (29.69)	21.370	2	<0,001
	**Systolic blood pressure**	110.00–160.00 126.00 (12.25)^***^	110.00–140.00 123.33 (6.61)^***^	60.00–140.00 116.56 (14.06)^***^	5.883	2	0,005
	**Diastolic blood pressure**	50.00–100.00 74.17 (12.25)	60.00–90.00 76.17 (7.39)^**^	60.00–110.00 77.59 (9.24)^***^	0.879	2	0,420

Source: own research; *p <0.05; **p <0.01; ***p <0.001.

In all cases the results of post-hoc tests were statistically insignificant (p > 0.1), no differences between the individual subgroups were shown using either Tukey’s HSD test or Dunnet’s T3 test (some of the compared groups showed heterogeneous variance of the selected measurements; in Levene’s test p < 0.05).

In the Stroop Test and Trail Making Test, apart from the time and number of correct answers, the number of mistakes made by patients was also checked. Due to the small number of errors in all groups, these variables were more ordinal than quantitative in the sample and were therefore checked using the non-parametric Kruskal–Wallis test. Similarly, as in the case of time and efficiency of tasks, no significant differences in errors made were observed between patients from the three groups (p > 0.05).

The groups differed in average values of such MS components as waist circumference, body weight, BMI, HDL and TG (p < 0.001) and systolic pressure (p < 0.01), there were no differences in glucose and diastolic pressure. Post-hoc tests showed that these differences occurred only between MSwithDI and MSw/oDI groups and SH, almost in every case with p < 0.01 significance, the exception was systolic pressure, where in Tukey’s HSD test with homogeneous variants the difference between SH and MSwithDI group was greater (p = 0.003) than between SH and MSw/oDI (p = 0.043).

**Table 3** presents the results of comparing the effects of cognitive performance measurement in patients undergoing dietary intervention. Only in this subgroup the improvement of cognitive performance was observed. After a change in dietary behavior in patients with schizophrenia with concomitant metabolic syndrome, an improvement in Stroop Test time was observed in the 3rd attempt (3.38 s, p < 0.05) and a decrease in Point Merging Test time in the 2nd attempt (16.78 s, p < 0.01). Moreover, in the second measurement patients from this group showed higher verbal fluency, on average 2.10 more animal names (p < 0.01) and 1.07 more acute subjects (p < 0.01). The working memory was also improved, resulting in a greater number of digits repeated directly (on average by 0.8 words more; p = 0.001) and in total (on average by slightly more than one digit more; p < 0.05).

**Table 3:** Comparison of results of cognitive function measurement before and after dietary intervention (MSwithDI group).

Tools	Task	Difference in mean values	Standard error in mean difference (SE)	95% confidence interval for mean difference (CI)		Student’s t-test		
				Bottom limit	Upper limit	t	df	P
**Stroop Test**	**Trial 1—time [s]**	−0.01	0.72	−1.49	1.47	−0.010	29	0.992
	**Trial 2—time [s]**	0.70	1.02	−1.40	2.79	0.679	29	0.502
	**Trial 3—time [s]**	3.38	1.60	.11	6.66	2.113	29	0.043
**Trail Making Test**	**Test A [s]**	0.14	2.81	−5.62	5.90	0.049	29	0.961
	**Test B [s]**	16.78	5.46	5.61	27.94	3.072	29	0.005
**Verbal Fluency Test**	**Animals**	−2.10	0.70	−3.53	−.67	−2.999	29	0.006
	**Objects starting with letter “K”**	−0.47	0.44	−1.36	.43	−1.064	29	0.296
	**Sharp objects**	−1.07	0.38	−1.85	−.28	−2.782	29	0.009
**Digit Span**	**Forward**	−0.80	0.21	−1.23	−.37	−3.788	29	0.001
	**Backward**	−0.23	0.29	−0.83	0.36	−0.804	29	0.428
	**All**	−1.03	0.42	−1.90	−0.17	−2.448	29	0.021

Source: own research.

In comparison, after 3 months in the group with MS without dietary intervention the level of performance of every cognitive test remained at the same level (**Table 4**).

**Table 4:** Comparison of cognitive function measurement results 3 months after the first measurement in patients with schizophrenia and co-occurring metabolic syndrome.

Tools	Task	Difference in mean values	Standard error in mean difference (SE)	95% confidence interval for mean difference (CI)		Student’s t-test		
				Bottom limit	Upper limit	t	df	P
**Stroop Test**	**Trial 1—time [s]**	1.51	0.90	−0.34	3.37	1.675	28	0.105
	**Trial 2—time [s]**	1.31	0.86	−0.46	3.07	1.517	28	0.141
	**Trial 3—time [s]**	−.022	1.94	−4.20	3.75	−0.115	28	0.909
**Trail Making Test**	**Test A [s]**	−0.20	1.68	−3.63	3.24	−0.117	28	0.908
	**Test B [s]**	4.75	5.21	−5.93	15.42	0,911	28	0.370
**Verbal Fluency Test**	**Animals**	0.45	0.61	−0.79	1.69	0.741	28	0.465
	**Objects starting with letter “K”**	−0.24	0.50	−1.27	0.79	−0.480	28	0.635
	**Sharp objects**	−0.14	0.47	−1.11	0.83	−0.292	28	0.773
**Digit Span**	**Forward**	0.11	0.23	−0.37	0.57	0.451	28	0.655
	**Backward**	−0.35	0.21	−0.78	0.09	−1.625	28	0.115
	**All**	−0.31	0.33	−0.98	0.36	−0.952	28	0.349

Source: own research.

Furthermore, some differences were observed between measurements I and II of cognitive functions in SH group (patients with schizophrenia without MS). In the second measurement these patients achieved significantly worse results in the first attempt of the Point Merging Test—on average by 3.69 s (p < 0.05) they also mentioned 1.42 fewer animal names (p < 0.05) and 0.83 fewer acute objects (p < 0.1). There was also a weak tendency to extend the time of performing the Stroop Test in the 1st sample by 1.04 s on average. Detailed information about the results of cognitive function measurement in the initial measurement and after 3 months in patients without MS is presented in **Table 5**.

**Table 5:** Comparison of results of cognitive function measurement 3 months after the first measurement in patients with schizophrenia without metabolic syndrome.

Tools	Task	Difference in mean values	Standard error in mean difference (SE)	95% confidence interval for mean difference (CI)		Student’s t-test		
				Bottom limit	Upper limit	t	df	p
**Stroop Test**	**Trial 1—time [s]**	−1.04	2.92	0.60	−2.27	−1.745	23	0.094
	**Trial 2—time [s]**	1.06	7.50	1.53	−2.11	0.693	23	0.495
	**Trial 3—time [s]**	−0.92	10.44	2.13	−5.33	−0.433	23	0.669
**Trail Making Test**	**Test A [s]**	3.69	8.38	1.71	0.16	2.159	23	0.041
	**Test B [s]**	−0.73	16.97	3.46	−7.90	−0.210	23	0.835
**Verbal Fluency Test**	**Animals**	1.42	2.84	0.58	0.22	2.442	23	0.023
	**Objects starting with letter “K”**	0.17	2.10	0.43	−0.72	0.389	23	0.701
	**Sharp objects**	0.83	2.06	0.42	−0.04	1.985	23	0.059
**Digit Span**	**Forward**	−0.25	1.11	0.23	−0.72	−1.100	23	0.283
	**Backward**	0.21	1.10	0.23	−0.26	0.926	23	0.364
	**All**	−0.21	1.84	0.38	−0.99	−0.554	23	0.585

Source: own research.

Changes between the first and second measurement were observed not only in the speed and effectiveness of the solved tasks, but also in the number of errors made in them. In the group of patients with dietary intervention in the second measurement the number of mistakes made in the Stroop Test in the second sample (Z = −2.504; p = 0.012) and third sample (Z = −3.393; p = 0.001) decreased, although an increase in the correctness of task execution in the last sample was also observed in patients without MS (Z = −2.173; p = 0.030). In this group, the number of errors made during the Trail Making Test in the first attempt (Z = −2,000; p = 0.046) also improved, furthermore in this test, the number of errors made by the control group of patients with MS was also lower, and the improvement was made in the second attempt (Z = −2.328; p = 0.020). It cannot be excluded that some intergroup differences between the two measurements may be due to the learning effect.

Both groups of patients with MS did not differ significantly in the distribution of basic sociodemographic features such as gender, age and level of education, but it was checked whether the relationships between these variables and cognitive performance were similar in all groups. In the initial measurement, very similar relationships between these characteristics were observed both in patients with MS for whom a dietary intervention was planned and in the comparative group. It was shown, for example, that with age the number of errors in the Stroop Test increased in both groups (in task 2 rho = 0.363; p = 0.049 in MSwithDI, and a stronger correlation was observed in the MSw/oDI group: rho = 0.687; p < 0.001, in task 3 rho = 367; p = 0.046 and rho = 0.350; p = 0.058). The time of task execution also increased (e.g. in task three in MSwithDI group rho = 0.446; p = 0.013 and in MSw/oDI group rho = 0.491; p = 0.006). In the MSwithDI group a significant increase of Test B score with age was noted (rho = 0.532; p = 0.002), whereas in the MSw/oDI group this relation remained at the level of weak tendency (rho = 0.313; p = 0.092). In both groups the number of digits repeated backwards decreased with increasing age, although in the MSwithDI group the number of digits was higher (rho = −0.439; p = 0.006) than in MSw/oDI (rho = −0.338; p = 0.068). In both groups, however, there were no relationships between age and verbal fluence.

The level of education was almost equally significant in the number of mistakes made in the Stroop Test in the last task (rho = 0.446; p = 0.013 in the MSwithDI group and rho = 0.491; p = 0.006 in the group without intervention), and qualitatively a slightly different meaning for the time of the second task—although in the MSwithDI group fully significant (rho = −0.489; p = 0.006) and in the MSw/oDI group at the level of statistical tendency (rho = −0.352; p = 0.056). Similarly, a decrease in the performance quality of the Trail Making Test was observed, with a significant decrease in the MSwithDI group (rho = −0.558; p = 0.002) and a trend in the MSw/oDI group (rho = −0.343; p = 0.064), while in Test A only among patients undergoing dietary intervention (rho = −0.511; p = 0.004). In both groups verbal fluence in the “Objects starting with K” sample increased moderately as education increased (successively rho = 0.438; p = 0.015 in MSwithDI I rho = 0.518; p = 0.003 in MSw/oDI), although only in the non-interventional group weak correlation was also observed in the “animals” sample. In case of digit repetition, among patients from the MSwithDI group the number of digits increased slightly (but not statistically significant) with the increase in education (rho = 0.353; p = 0.056) and in MSw/oDI the number of words spoken backwards significantly increased (rho = 0.525; p = 0.003).

In the case of gender differences in the first measurement, occasional differences of minor importance were noticed. It turns out that only among patients from the MSw/oDI group in the measurement of verbal fluence in the “Animals” sample, men reached slightly more (but statistically insignificant) points than women (Z = −0.1856; p=0.063), moreover, in the digit repetition test in both groups men were able to repeat more digits backwards, while in the MSwithDI group this gender difference was fully significant (Z = −2.004; p = 0.045) and in MSw/oDI it was weaker—at tendency level (Z = −1.783; p = 0.075).

Analyzing the importance of sociodemographic variables in the view of results obtained in the second measurement—after a dietary intervention or after a 3-month interval—very similar results were obtained. This additional analysis suggests that just as the compared groups of patients with MS did not differ significantly in the overall distribution of the most important sociodemographic characteristics, the importance of these characteristics in shaping cognitive performance in both measurements was very similar. It can therefore be concluded that additional sociodemographic factors did not play a significant role in the picture of the phenomenon under study.

## Discussion

There are studies showing that metabolic syndrome, and especially obesity, is associated with the emergence of cognitive disorders in people with mental illness. Individuals with obesity as a major constituent of MS may show deficits in executive functions (worse decision making, problems with planning, inhibition of reaction, reduced elasticity in action) and decreased memory function, speed of reaction, language skills or concept development (39–41). However, this study did not demonstrate that the metabolic syndrome itself is a cause of cognitive decline, as there were no statistically significant differences in cognitive function between patient groups before the introduction of dietary intervention. Similar results were obtained in the CATIE study, where no links between the metabolic syndrome and cognitive disorders in schizophrenia were found (42). Also other research teams did not show any relationship between BMI and cognitive functions (43, 44). Perhaps the reason for the above discrepancies in the relationship between metabolic syndrome and cognitive disorders is the fact that the individual components of the syndrome may rather be related to specific disorders of these functions. A different cause may be found in varying degrees of education, age of patients, or duration of disease in different studies quoted above. For example, it is known that the early onset of schizophrenia may be associated with worse cognitive function (45). The gender of the respondents may also influence the results, as the studies show that women are more likely to suffer from negative consequences of metabolic disorders (46), including cognitive dysfunctions resulting from obesity (47).

The observed improvement in cognitive function tests after dietary intervention seems to be a very interesting result in the context of a holistic approach to the treatment of schizophrenia. We have long known that the management of this disease cannot be limited to pharmacological treatment only. Patients should be encouraged to try to change their lifestyle. The literature on the subject shows that the lifestyle of people with schizophrenia is one of the most important factors which determine the emergence of coexisting chronic diseases. Researchers observed significantly more frequent smoking, alcohol consumption and excessive amounts of food, while at the same time lower physical activity in patients with coexisting somatic diseases. Increased energy supply is the main dietary risk factor of developing coexisting diseases (48). The CHANGE study showed that people with schizophrenia spectrum had higher intakes of saturated fats, sugar and alcohol. However, the amount of fish, vegetables and fruit consumed was insufficient. From the studied group (n = 428) only 10.7% of people used healthy eating patterns (49, 50). Dietary mistakes made by patients with schizophrenia may result from their lack of knowledge and inability to obtain any support in changing eating habits. In this study, the majority of patients who received a nutrition plan tried to follow it. These patients willingly decided to reduce their body weight and improve their eating habits. It is very important to provide education on the influence of the drugs used, on metabolism. It should be pointed out how to avoid side effects in the form of weight gain after the applied pharmacological treatment. The patient should know that the healing process is not only a systematic intake of medication, but also a change in lifestyle.

Clinical studies also show that brain-derived neurotrophic factor (BDNF) is one of the biomarkers associated with cognitive function. There is a significant relationship between MS and decreased BDNF level, which affects the plasticity of synapses. A low-energy diet may contribute to an increase in BDNF (51, 52). The introduction of a diet with calorie reduction of about 500 kcal and compliance with the diet resulted in the reduction of the components of MS, which may have contributed to the reduction of systemic inflammation and increase in the level of BDNF factor, which in turn could have a positive effect on cognitive deficits in schizophrenic patients. The final decision whether this was the mechanism of cognitive improvement will be the result of a study with the measurement of the level of BDNF during dietary intervention.

One of the elements used in the dietary plan was an increase in essential polyunsaturated fatty acids (PUFA). Many scientific studies point to the connection between the consumption of OMEGA-3 fatty acids and the treatment of mental disorders (53–56). More and more evidence suggests the occurrence of oxidative stress in schizophrenia, as the body is unable to balance the production of reactive oxygen species (ROS) and reactive nitrogen (RNS) produced from oxidative metabolism. Oxidative stress and impaired defense mechanisms may be associated with neurodevelopmental disorders, changes in brain structures, intensified negative symptoms or cognitive impairment (57). The supplementation of essential polyunsaturated fatty acids (PUFA) and alpha-tocopherol reduces the total glutathione concentration, whereas the administration of saturated fatty acids results in a significant decrease in gamma- and delta-tocopherol (58). MS is associated with chronic inflammation, and the supply of large amounts of saturated fatty acids from the diet contributes to an increase in the level of inflammation markers (59, 60). Lipid profile disturbances and obesity increase the risk of arterial atherosclerosis. Atherosclerosis may contribute to the occurrence of cognitive deficits. A significant disturbance of metabolic parameters may cause micro and macroscopic cerebrovascular changes, which may hinder the nervous system function and cause cognitive disorders in memory, executive functions or attention (61). There are also data on the effect of excessive fatty tissue on nervous tissue through neurochemical mediators, e.g. leptin (62). In this study, in patients with schizophrenia without metabolic syndrome, cognitive function deteriorated 3 months after the first visit, which may result from lack of nutritional awareness and persistence in their previous unhealthy eating habits, which negatively affected cognitive functions, causing their gradual deterioration. It is important to emphasize the importance of awareness of patients participating in the study, who subjectively assessed their eating habits as inadequate. At least some people with schizophrenia are aware that they lead an unhealthy lifestyle (63). Therefore, when working with patients, both psycho-education regarding proper nutrition and work on motivation to change their lifestyle should be undertaken. A small group of patients is a limitation of the study, but further work with a larger cohort is planned.

The lack of statistically significant differences between the MSwithDI and MSw/oDI groups speaks in favor of methodological correctness and the possibility to regard the group without intervention as a control for experimental measurement. On the other hand, the lack of differences between the two groups of patients with MS and the subgroup of patients with schizophrenia alone, suggests that the occurrence of metabolic syndrome does not have to be associated with a deterioration of cognitive performance in patients with schizophrenia.

The obtained results did not confirm the thesis that cognitive function in patients with schizophrenia and co-morbid metabolic syndrome is worse than in patients without metabolic syndrome. However, it was possible to demonstrate the importance of dietary intervention in the pursuit of cognitive function improvement. Understanding the relationship between diet and cognitive ability in schizophrenia may contribute to the development of therapeutic strategies that will not only avoid the development of MS, but also improve the level of functioning of people with schizophrenia. Change of eating habits may be a significant element of a holistic approach to the problems of treatment of schizophrenia.

## Limitations

The project had a number of limitations that may have had the potential to obstruct the research procedure and the final results. The study was of a correlative nature, which did not allow to determine the cause–effect relationships, so it was not known what was the primary and secondary variable. Discussing eating habits and evaluating them subjectively may have sometimes been accompanied by a desire to present oneself in a favorable light. Attempts have been made to ensure that the person assessing cognitive function was not informed about the patients’ affiliation to a given group, but it was not always possible to achieve this because of the differences in body weight between groups.

## Data Availability Statement

The datasets generated for this study are available on request to the corresponding author.

## Ethics Statement

The studies involving human participants were reviewed and approved by Bioethics Committee of the Pomeranian Medical University in Szczecin (Resolution No. KB-0012/108/14). The patients/participants provided their written informed consent to participate in this study.

## Author Contributions

KA: project design, data collection, database preparation, preparation of patients’ nutritional plans, statistical calculations. AM: text editing, translation into a foreign language. MM: the choice of statistical tools. JS: verification of the content and approval of the final version of the manuscript. JK-M: project concept, project design, choice of statistical tools, critical content verification, choice of journal.

## Funding

The project was financed under the program of the Ministry of Science and Higher Education under the name “Regional Initiative of Excellence” for the years 2019–2022, project number 002/RID/2018/19, funding amount PLN 12,000,000. This study was partly supported by the Young Researcher project at the Pomeranian University in Szczecin (MB-312-199/16/17) and by the Clinic and Chair of Psychiatry at the Pomeranian University in Szczecin, Science Stimulation Fund.

## Conflict of Interest

The authors declare that the research was conducted in the absence of any commercial or financial relationships that could be construed as a potential conflict of interest.

## Abbreviations

MS, metabolic syndrome; TMT, Trail Making Test; SH—patients with schizophrenia without metabolic syndrome; MSwithDI, patients with schizophrenia, metabolic syndrome and dietary intervention; MSw/oDI, patients with schizophrenia, metabolic syndrome, without dietary intervention; PUFA, polyunsaturated fatty acids; ROS, reactive oxygen species; RNS, reactive nitrogen species; CNS, central nervous system
